# Is Pearson’s correlation coefficient enough for functional connectivity in fMRI?

**DOI:** 10.1162/IMAG.a.1052

**Published:** 2025-12-08

**Authors:** Hecheng Jin, Julian S.B. Ramirez, Kyoungseob Byeon, Brian E. Russ, Arnaud Falchier, Gary Linn, Gregory Kiar, Charles E. Schroeder, Joshua T. Vogelstein, Michael P. Milham, Ting Xu

**Affiliations:** Child Mind Institute, New York, NY, United States; Nathan Kline Institute, New York, NY, United States; Nash Family Department of Neuroscience and Friedman Brain Institute, Icahn School of Medicine at Mount Sinai, One Gustave L. Levy Place, New York, NY, United States; Department of Psychiatry, New York University at Langone, New York, NY, United States; Columbia University, New York, NY, United States; Johns Hopkins University, Baltimore, MD, United States

**Keywords:** fMRI, functional connectivity, nonlinear dependencies, Pearson’s correlation coefficient, Multiscale Graph Correlation

## Abstract

Functional connectivity (FC) is commonly defined as the temporal coincidence of neurophysiological events, often quantified by the statistical dependency among signals from different brain regions and measured by Pearson’s correlation coefficient in fMRI. However, Pearson’s r captures only linear dependencies, potentially overlooking nonlinear interactions. Recently, Multiscale Graph Correlation (MGC) was introduced to measure statistical dependencies of both linear and nonlinear relationships across multiple scales, offering an “optimal scale” at which such dependencies can be inferred. In this study, we systematically compared FC measurements by Pearson’s r and MGC across datasets, evaluating their reliability, sensitivity to data quantity, and ability to capture distinct experimental conditions (deeper anesthesia in macaques) and brain-behavior association. Results showed highly similar spatial connectivity patterns and strong alignment between Pearson’s r and MGC for within-network FC, where optimal scales were frequently global. However, local optimal scales emerged between networks, suggesting the presence of nonlinear dependencies of FC. Reliability was higher for Pearson’s r overall, but both measurements improved as the quantity of data increased. Notably, MGC revealed variability in the optimal scales under altered brain states in deeper anesthesia, highlighting its potential for detecting local-scale dependencies across states. Despite these advantages, MGC required greater computational resources and did not outperform Pearson’s r in detecting brain-behavior associations. Consequently, Pearson’s r remains a sufficient and reliable measure for many standard applications, whereas MGC can offer more nuanced insights in scenarios where nonlinear dynamics are of particular interest. Researchers should, therefore, balance the potential gains from MGC against its added complexity and computational cost when selecting methods to quantify FC.

## 1 Introduction

Functional connectivity (FC) has been defined as statistical dependencies of temporal neurophysiological events (Friston, 1994), reflecting the synchronized activity and communication within and across segregated brain regions. FC serves as a key measure for understanding how different brain regions interact and coordinate, thereby revealing the network organization that supports cognitive functions and identifying changes associated with normal brain development, aging, and neurological or psychiatric conditions (Rubia, 2013; Zang et al., 2007; Zhang et al., 2021). The most conventional FC measure is quantified using Pearson’s correlation coefficient (i.e., Pearson’s r), which focuses on linear relationships and typically assumes that interactions between two regions are stationary in functional magnetic resonance imaging (fMRI) studies. This assumption—linear relationship of fMRI blood oxygenation level-dependent (BOLD) signals between brain regions—can be advantageous for its simplicity, interpretability, and low computational cost. However, a growing body of work suggests that functional interactions may also unfold at multiple scales and involve more complex, potentially nonlinear relationships (Calhoun et al., 2014; Chang & Glover, 2010; Cohen, 2018; Hutchison et al., 2013). This highlights a notable limitation of Pearson’s r: its restriction to linear dependencies, which might not fully account for nonlinear dependencies of functional connectivity.

To address these limitations, advanced statistical approaches have been developed to capture complex dependencies, including causal indices, distance similarity, information theory, and spectral-based methods (Cliff et al., 2023), such as Distance Correlation (Dcorr) (Székely et al., 2007; Székely & Rizzo, 2009), Modified versions of Dcorr (Mcorr) (Székely & Rizzo, 2013), Heller-Heller-Gorfine’s test (HHG) (Heller et al., 2013), and Hilbert Schmidt Independence Criterion (HSIC) (Gretton & Györfi, 2010; Muandet et al., 2017). These methods aim to generalize the notion of dependence beyond linearity and address the limitations of traditional Pearson’s r measurement. Among them, Dcorr is a widely used method that extends conventional correlation by detecting both linear and nonlinear dependencies across arbitrary dimensions and structured data (Székely et al., 2007; Székely & Rizzo, 2009, 2013). However, its sensitivity can be limited in high-dimensional settings under typical sample-size conditions (Vogelstein et al., 2019). To further address these limitations, Multiscale Graph Correlation (MGC) has been proposed, offering a more comprehensive framework to capture complex relationships (Shen et al., 2020, 2024; Vogelstein et al., 2019).

MGC integrates the strengths of distance-based methods, evaluating dependencies across multiple scales to model linear and nonlinear dependencies in a generalizable manner. This enables MGC to detect both linear and nonlinear relationships effectively, even in high-dimensional data. Extensive simulations have demonstrated that MGC reliably detects a broad range of linear and nonlinear dependencies, often outperforming the above methods, including Dcorr, Mcorr, mutual information (MIC), etc., particularly in high-dimensional and nonlinear scenarios (Vogelstein et al., 2019). A key innovation of MGC is its ability to characterize the ‘optimal scale’ of a tested relationship. By evaluating the local linear relationships over multiple neighboring data points (i.e., ‘scale’) in the sample space, MGC identifies the scale at which the strongest dependency occurs as the ‘optimal scale’. The concept of the optimal scale is particularly important for FC analysis, as it reflects the scale (i.e., the number of time point samples in the distance space) at which fMRI signals from two brain regions exhibit statistical dependence across the distribution of the fMRI signals. When the optimal scale is detected at the largest possible scale (i.e., all time-point samples in fMRI signals), this is referred to as a ‘global’ scale, indicating that the observed dependency is consistent across the entire joint distribution of their fMRI signals between two brain regions. In contrast, a ‘localized’ optimal scale where the optimal dependence peaks at smaller neighborhood sizes suggests that the relationship is most prominent within more restricted subsets of the fMRI data space, reflecting more complex, potentially nonlinear interactions between two regions. Therefore, by capturing context-specific dependencies, MGC provides a nuanced, scale-sensitive framework for uncovering intricate dependencies in fMRI data, offering insights beyond those afforded by traditional linear metrics like Pearson’s r. Given the characteristic of FC analysis, where brain oscillations are not assumed to be purely linear dependencies across regions, applying MGC to investigate FC and quantify the optimal scale of fMRI signal allows for a detailed examination of the complexity of brain interactions.

In this study, we systematically investigated whether FC in diverse fMRI datasets is more accurately characterized by linear measures like Pearson’s r or by MGC’s multiscale approach. We applied both methods in multiple datasets (the Human Connectome Project (HCP) (Van Essen et al., 2013), the NKI-Rockland Sample (Nooner et al., 2012), and rhesus macaque scanned in awake and anesthetized states). We also assessed the test-retest reliability of each method, the effect of varying data quantity, and the efficacy of each approach in explaining behavioral variability (e.g., developmental age). Additionally, we focused on homotopic FC to determine whether MGC could reveal additional scale-dependent dependencies that Pearson’s correlation might overlook. This included comparisons of the optimal scale of homotopic FC across various amounts of time points in humans and state differences between awake and anesthetized states in macaques.

## 2 Methods

### 2.1 Datasets

#### 2.1.1 Human Connectome Project (HCP)

We used the HCP test-retest dataset, which contains 2 resting-state fMRI sessions (2 scans per session) repeated twice per individual (8 scans in total). The resting-state fMRI (rfMRI) data were acquired with TR = 720 ms, TE = 33.1 ms, FOV = 208 x 180 mm, resolution = 2.0 mm isotropic voxels, slice thickness = 2.0 mm, and 72 slices. The HCP studies were approved by the Washington University in St. Louis Institutional Review Board. Details of the dataset were reported previously in Glasser et al. (2013) and documented on the HCP website (https://www.humanconnectome.org/study/hcp-young-adult). The minimal-preprocessed data were further preprocessed in our prior study (Cho et al., 2021). There were 31 participants who completed all tasks and resting-state scans with low head motion (mean frame-wise displacement <0.25). We further excluded one more subject who had an inconsistency in the number of time points of resting-state scans.

#### 2.1.2 Nathan Kline Institute-Rockland sample (NKI-RS)

The NKI-RS dataset (Nooner et al., 2012) is publicly available through the 1000 Functional Connectomes Project (FCP, https://fcon_1000.projects.nitrc.org/indi/enhanced/). The rfMRI data were acquired using two protocols: (1) TR = 1400 ms, TE = 30 ms, FOV = 224 x 224 mm, resolution = 2.0 mm isotropic voxels, slice thickness = 2.0 mm, and 64 slices; (2) TR = 645 ms, TE = 30 ms, FOV = 222 x 222 mm, resolution = 3.0 mm isotropic voxels, slice thickness = 3.0 mm, and 40 slices. The NKI-RS received Institutional Review Board approval from the NKI (Phase I #226781, Phase II #239708) and Montclair State University (Phase I #000983A, Phase II #000983B). The dataset was detailed in Nooner et al. (2012). In our study, we selected the developmental sample from childhood to early adulthood (194 subjects, 327 scans, age = 13.34 ± 3.65, 6–22 years) included in our analysis.

#### 2.1.3 Macaque dataset

The macaque dataset consisted of 3 rhesus macaques (*M. mulatta*). They were scanned on a Siemens Tim Trio scanner (3.0 T) at the NKI with an 8-channel surface coil. The rfMRI data were acquired with TR = 2000 ms, TE = 16.6 ms, FOV = 96 x 96 mm, resolution = 1.5 x 1.5 x 2.0 mm, slice thickness = 2.0 mm, and 32 slices. Each macaque was scanned in both awake and anesthetized states. During an anesthetized session, the animals were sedated for intubation with ketamine (10 mg/kg) followed by isoflurane to maintain a sedation state. Concentrations of 0.75%, 1.0%, 1.5%, or 2.0% isoflurane were given either in ascending (starting at 0.75%) or descending (starting at 2.0%) order with pseudo-randomization. Two 8-min (240 time points) scans were collected at each level between switching concentrations. Four to 12 sessions in total were acquired for each macaque. During the awake session, the macaques were chaired in an MR-compatible monkey chair with a head-fixation to control the head motion. The details of the acquisition were reported in our previous paper (Xu et al., 2018). All procedures were approved in advance by the Institutional Animal Care and Use Committee (IACUC) of the NKI.

#### 2.1.4 Functional MRI preprocessing

The HCP test-retest rfMRI data, processed with the HCP minimal preprocessing pipeline (Glasser et al., 2013; Marcus et al., 2013), were acquired from the HCP website. Subsequently, further preprocessing was applied, including nuisance regression (Friston’s 24 head motion parameters, mean signals from white matter, cerebrospinal fluid, and global signal) and bandpass filtering (0.01–0.1 Hz) (Cho et al., 2021). The preprocessed data were then projected to the cortical surface, smoothed with FWHM = 6 mm, and averaged using the Glasser360 (Glasser et al., 2016) and Schaefer200 (Schaefer et al., 2018) parcellations.

The NKI-RS rfMRI data were processed using the fMRIPrep (Esteban et al., 2019) minimal preprocessing pipeline, followed by the XCP (Mehta et al., 2024) imaging pipeline. The fMRIPrep pipeline’s minimal preprocessing steps included motion correction, field unwarping, normalization, bias field correction, and brain extraction. The output of fMRIPrep was then processed through XCP, which involved despiking, bandpass filtering (0.01–0.1 Hz), regression of confounds (36 head motion parameters, global signal, CompCor), smoothing (FWHM = 6 mm), and parcellating the time series according to the Glasser360 (Glasser et al., 2016) atlas.

The Macaque rfMRI data preprocessing included temporal compressing (3dDespike), motion correction, 4D global scaling, nuisance regression (Friston’s 24 head motion parameters, mean signals from white matter, cerebrospinal fluid), and bandpass filtering (0.01–0.1 Hz). The preprocessed data were then projected to the mid-cortical surface, smoothed with FWHM = 3 mm, and averaged using the Markov et al. (2014) parcellations.

#### 2.1.5 FC measured by Pearson’s r and MGC

Pearson’s correlation coefficient (Pearson’s r) measures the linear relationship between two datasets. Pearson’s r was calculated using the *pearsonr* from the SciPy Python package (https://docs.scipy.org/doc/scipy/reference/generated/scipy.stats.pearsonr.html).

**Fig. 1.**
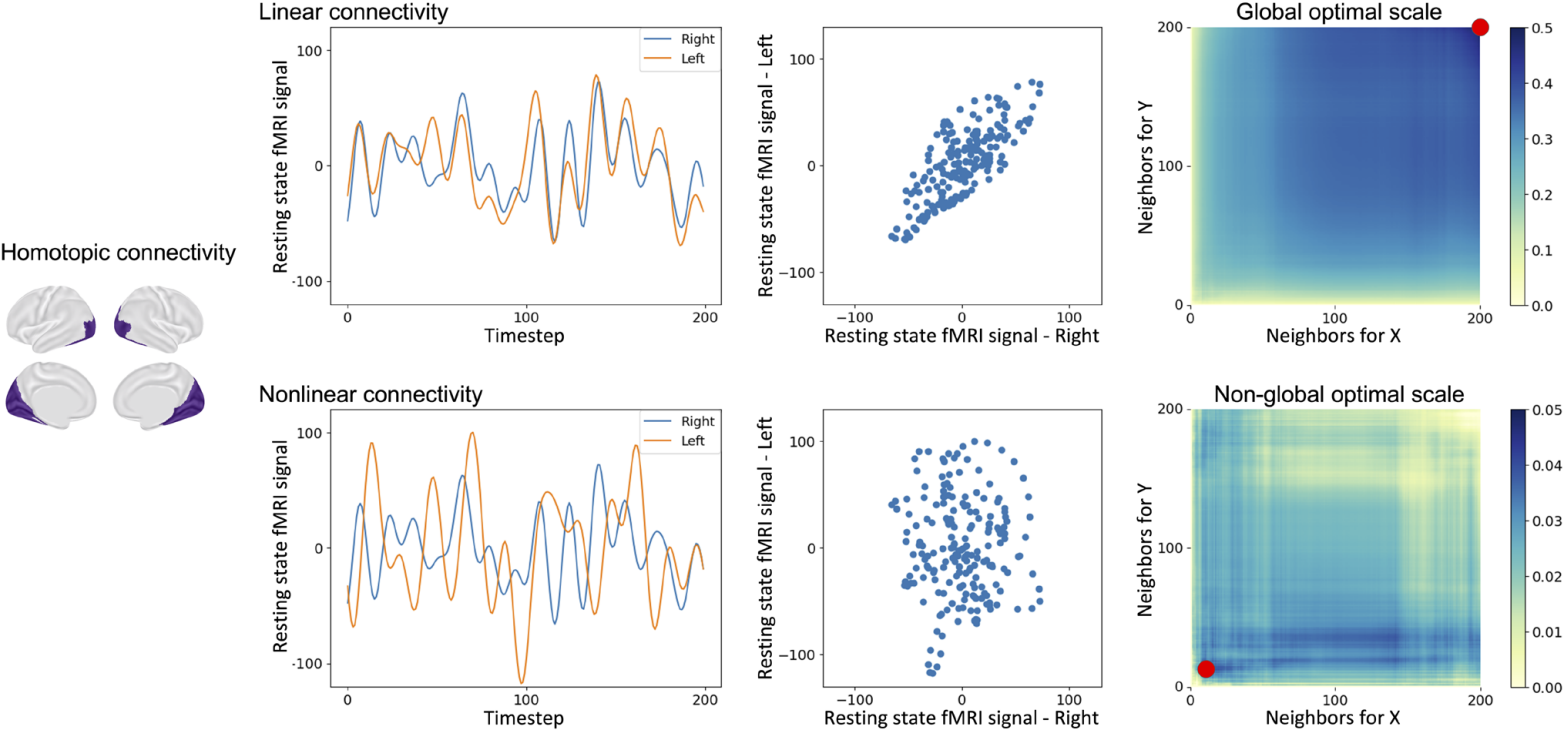
Example demonstrating homotopic connectivity measured by MGC, depicting linear and nonlinear time series along with their corresponding local correlation maps.

Multiscale Graph Correlation (MGC) provides two indices: the MGC statistic and ‘optimal scale’. MGC statistic computes a coefficient to assess the strength of statistical dependence between two signals from brain regions. The optimal scale, ranging from 0 to the sample size (i.e., the full length of the time points), suggests the number of fMRI signal samples that achieve the maximal correlation, which identifies potential nonlinear dependency relationships when the optimal scale is non-global or local (i.e., less than the full length of the time point samples). For interpretability and comparability across datasets, all optimal scale values in this study were normalized by dividing by the total number of time points, yielding values between 0 and 1. A larger optimal scale close to 1 suggests that the dependence spans most or all of the scan time points (i.e., global), whereas a smaller, non-global (i.e., local) scale indicates that the strongest dependency is confined to a more localized subset of time point samples, indicating the presence of nonlinear relationships (Fig. 1). Importantly, while linear dependencies have been theoretically shown to yield a global scale (Vogelstein et al., 2019), the converse is not necessarily true: global scale does not guarantee linearity. On the other hand, a local (non-global) optimal scale is clearly indicative of nonlinearity. Therefore, observing local optimal scales in FC supports the presence of nonlinear relationships. As Pearson’s r evaluates zero-lag instantaneous dependencies, we applied the time-series MGC (Shen et al., 2024) with lag = 0 to ensure a fair and consistent comparison between the two approaches. Specifically, MGC statistic and optimal scale were calculated using the *MGCX (lag* *=* *0)* from the *hyppo* Python package (https://hyppo.neurodata.io/api/generated/hyppo.time_series.mgcx).

Additionally, we used dynamic time warping (DTW) as an alternative nonlinear measure to provide a comparison to MGC (Linke et al., 2020; Meszlényi et al., 2017; Sakoe & Chiba, 1978). Specifically, DTW was calculated using the *dtw* function from the DTAIDistance package (https://pypi.org/project/dtaidistance/) (Meert et al., 2025). The computational cost (CPU runtime) was evaluated and summarized in Supplementary Table S2.

Pearson’s r was also utilized to calculate the spatial similarity between FC maps derived by Pearson’s r and MGC. To calculate the similarity between FC maps, we only considered the magnitude in the Pearson FC, using absolute value to align with MGC’s focus on the magnitude of relationships rather than their directions.

### 2.2 Reliability analysis

The discriminability index assesses the extent to which measurements can differentiate between subjects, based on the assumption that repeated measurements of the same subject should exhibit greater similarity than those between different subjects. Discriminability testing (Bridgeford et al., 2021) was conducted using the one-sample discriminability test from the *hyppo* Python package (https://hyppo.neurodata.io/api/generated/hyppo.discrim.discrimonesample).

Table 1 provides an overview of our analyses to assess the utility and robustness of MGC. In Study 1, we compared MGC-measured FC with traditional Pearson’s r to understand the linear and nonlinear FC profiles and their spatial similarity with Pearson FC. Additionally, we evaluated the stability of these two methods across varying data amounts. In Study 2, we established the reliability of MGC-FC by calculating its discriminability and examining the impact of different amounts of data on both MGC and Pearson FC. Building upon these comparisons and reliability assessments, in Study 3-4, we examined whether MGC could reveal scale-dependent nonlinear homotopic FC that are not detectable with traditional Pearson’s correlation. Study 3 utilized both human and macaque data, comparing optimal scales of homotopic FC in humans and state differences (awake versus anesthetized) in macaques. Study 4 used the linear mixed-effects model to compare the age-related changes in FC profiles between the two methods.

**Table 1.** Study design, datasets, and analyses.

Study	Dataset and amount of data per subject	Analyses
Study 1: Comparison of FC pattern measured by Pearson’s r and MGC	HCP, 30 subjects • 200 time points • 400 time points • 800 time points	1. Examined the parcel-wise (Glasser360) FC using MGC and compared its spatial distribution of coefficients and optimal scale with the traditional FC measured by Pearson’s r (Fig. 2B). 2. Summarized MGC and optimal scale at the network level defined by Yeo et al. (2011) (Fig. 2C). 3. Examined the stability of Pearson’s r, MGC, and optimal scale as a function of the amount of data per subject. Specifically, we analyzed subsets per subject with 200 time points (8 subsets in total per subject), 400 time points (averaging FC across 2 scans, each consisting of 200 time points, 4 subsets in total per subject), and 800 time points (averaging FC across 4 scans, each consisting of 200 time points, 2 subsets in total per subject) (Fig. 3).
Study 2: Reliability of MGC FC	HCP, 30 subjects • test-retest subsets (400 time points in total per subset) • test-retest subsets (800 time points in total per subset)	1. Calculated the test-retest reliability (i.e., discriminability) of Pearson’s r, MGC, and optimal scale for 400 time points versus 400 time points (averaging FC across 2 scans, each consisting of 200 time points) and 800 time points versus 800 time points (averaging FC across 4 scans, each consisting of 200 time points) (Fig. 4). 2. We repeated the analyses using two parcellations (Glasser360 and Schaefer200) (Supplementary Fig. S2 and Supplementary Table S1).
Study 3: Homotopic FC using MGC	HCP, 30 subjects • 200 time points • 400 time points • 800 time points NKI-RS, 85 subjects • TR = 1400 ms, 402 time points • TR = 645 ms, 402 time points	1. Examined how homotopic FC and optimal scale, measured by Pearson’s r and MGC, vary across increasing data quantities using the HCP dataset. Specifically, we calculated homotopic FC across varying acquisition durations (i.e., the first 200, 400, and 800 time points for each of 8 resting-fMRI scans) (Fig. 5). 2. Comparing homotopic FC measured by Pearson’s r, MGC, and Dynamic Time Warping (DTW) across increasing data quantities (200, 400, and 800 time points for each of 8 resting-fMRI scans) (Supplementary Fig. S3) 3. Comparing homotopic FC measured by Pearson’s r, MGC across different scan acquisitions (TR = 1400 ms versus TR = 645 ms, 402 time points per subset for each individual) using the NKI-RS dataset (Supplementary Fig. S4)
	NKI-Macaque, 3 subjects • 4-12 scans per animal	4. To examine whether MGC can indicate awake and anesthetic states, we compared homotopic FC measured by Pearson’s r, MGC, and optimal scale across different anesthetic states in macaque monkeys (240 time points per scan) (Fig. 6).
Study 4: Brain-behavioral relationship using MGC	NKI-RS,194 subjects • 1-2 scans (404 time points) per subject	1. To verify if Pearson’s r and MGC FC captured similar age effects, we explored the age-related changes of homotopic FC measured by Pearson’s r and MGC (sex was included as a covariate) (Supplementary Fig. S6).

## 3 Results

### 3.1 Pearson’s r and MGC captured similar FC profiles

**Fig. 2.**
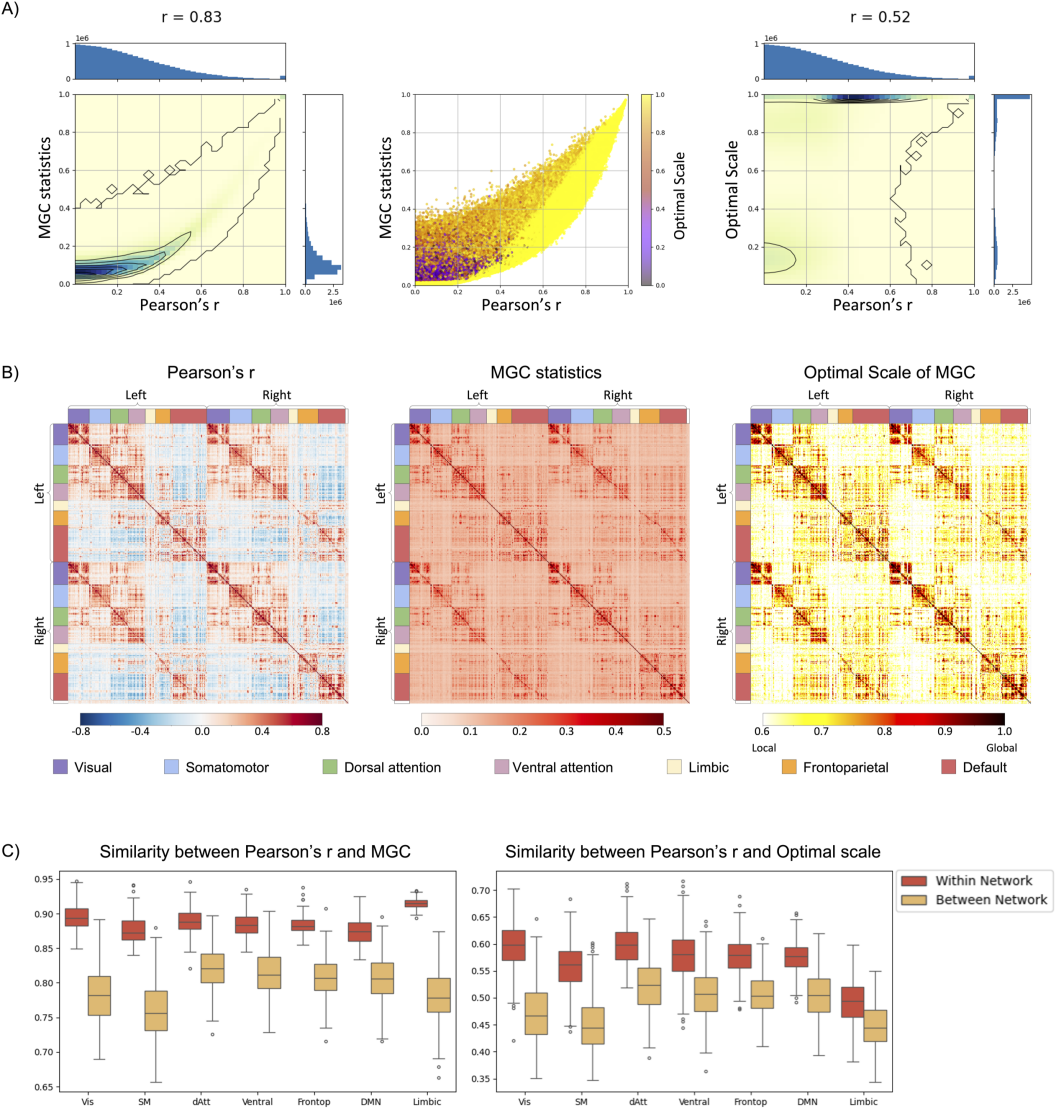
FC measured by Pearson’s r, MGC statistics, and its optimal scale. (A) Distributions of individual FC values as measured by Pearson’s r, MGC statistic, and optimal scale: a density plot of Pearson’s r and MGC statistic with contour lines and histograms on the edges (left), scatter plot colored by optimal scale (middle), and a density plot of Pearson’s r and MGC optimal scale (right). (B) Group-level FC matrix measured by Pearson’s r, MGC statistics, and its optimal scale. (C) Individual-level similarity between Pearson’s r, MGC statistics, and optimal scale: Pearson’s r exhibited a strong similarity with MGC statistics and optimal scale, particularly for the FCs estimated within networks.

We measured FC using Pearson’s r and MGC, comparing their spatial patterns in Study 1. At the individual level, we observed a strong positive relationship between Pearson’s r and the MGC statistic (r = 0.83; Fig. 2A left). Notably, there were no observations in which Pearson’s r was extremely low while the MGC statistic was high (Fig. 2A middle), indicating that weak linear associations in our resting-state dataset are generally accompanied by low overall dependence. This supports the notion that most strong relationships in the data are linear or approximately linear in nature. Furthermore, 51.4% of FC values exhibited optimal scales greater than 0.8 (Fig. 2A right), implying that a large proportion of FCs tends to be characterized at near-global scales, with 1 representing the global maximum.

At the group-level (Fig. 2B), the spatial distribution of FC identified by MGC also closely aligned with that obtained using Pearson’s r (Fig. 2B, left and middle panels, correlation = 0.938, p < 0.001). Regions with higher Pearson’s r values also exhibited higher MGC statistics, particularly for the within-network FC. Interestingly, the optimal scale of MGC suggested that FC relationships vary in the scale at which dependencies are strongest. Only the within-network FC (diagonal submatrices) exhibited the global optimal scale across the entire time series (Fig. 2B right), suggesting the consistent dependencies extend across the full sample space of fMRI signal between these regions. In contrast, between-network FC (off-diagonal submatrices) showed more localized optimal scale (i.e., less than 1), indicating that dependencies were restricted within subsets of the data. This implies the presence of nonlinear relationships between networks captured by MGC. Comparing Pearson’s r and optimal scale, FC with higher Pearson’s r values tended to correspond with higher optimal scales (Fig. 2B, left and right panels, correlation = 0.923, p < 0.001).

Comparing Pearson’s r and MGC at the network level (Fig. 2C and Fig. S1), a higher similarity was observed within each of the 7 networks defined by Yeo et al. (2011). The average similarity between Pearson’s r and MGC statistics within networks was 0.888 ± 0.021. In contrast, the similarity between-networks FC was consistently lower at 0.796 ± 0.042. Similarity to Pearson’s r was notably lower for the optimal scale than MGC statistics. Nonetheless, we again found lower similarity to Pearson FC between networks (0.487 ± 0.054) than within networks FC (0.568 ± 0.052).

**Fig. 3.**
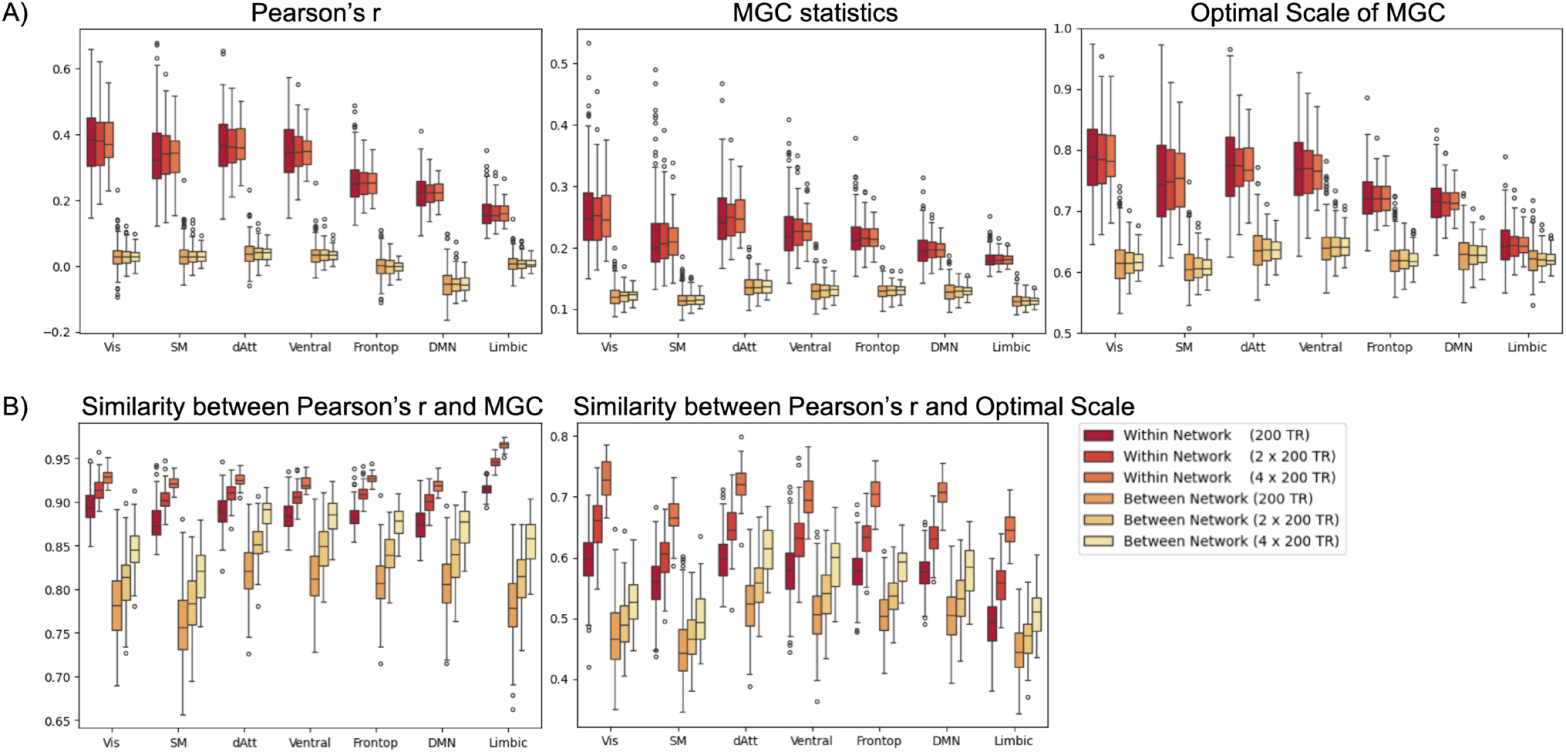
Comparison of FC measured by Pearson’s r, MGC statistics, and optimal scale across different data quantities per subject: 200 time points (1 scan), 400 time points (averaging FC of 2 scans, each consisting of 200 time points), and 800 time points (averaging FC of 4 scans, each consisting of 200 time points). (A) Pearson’s r, MGC statistics, and optimal scale of FC across data quantities. Higher Pearson’s r, MGC statistics, and optimal scales were observed within networks compared to those between networks. (B) Similarity between Pearson’s r, MGC statistics, and optimal scale: Higher similarities were consistently observed within networks, increasing with data quantities.

Furthermore, we examined the impact of the amount of data in measuring FC using MGC. As detailed in Study 1, we calculated Pearson’s r and MGC statistics for each scan (200 time points) per subject. To assess the effect of data quantity, Pearson’s r and MGC metrics were averaged across 2 scans (400 time points in total) and 4 scans (800 time points in total). As shown in Figure 3A, both Pearson’s r and MGC consistently exhibited stronger connectivity within networks compared to those between networks, regardless of data quantity. More importantly, as the amount of data increased, similarities between Pearson’s r and MGC statistics, as well as between Pearson’s r and the optimal scale, increased (Fig. 3B), indicating a stronger convergence between the two methods with more data.

### 3.2 Reliability increased with the amount of data measured by Pearson’s r and MGC

**Fig. 4.**
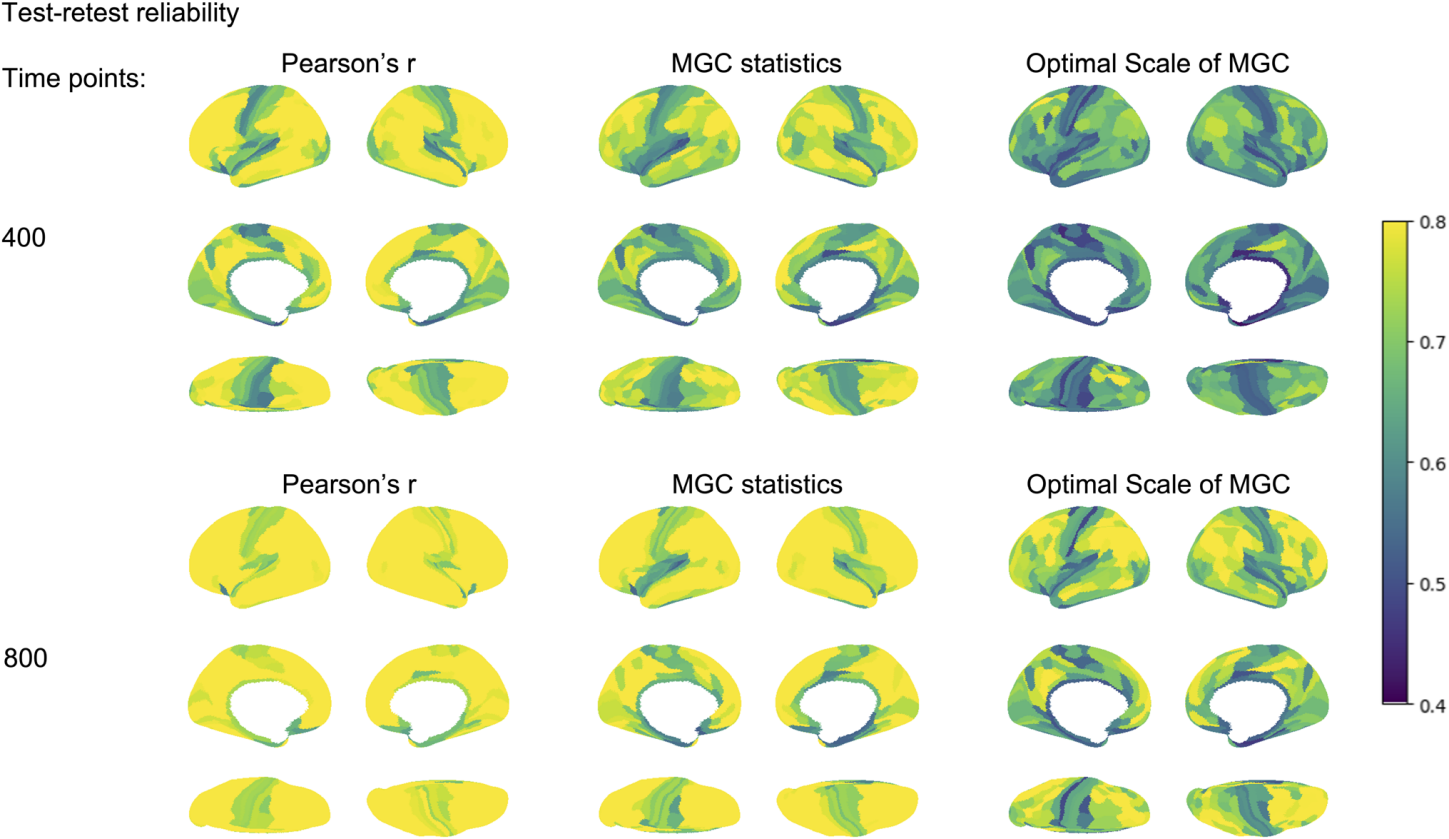
Test-retest reliability for the HCP dataset in Study 2, using 400 time points and 800 time points on Glasser360 (Glasser et al., 2016) parcellations.

After comparing FC measured by Pearson’s r and MGC in Study 1, we assessed the test-retest reliability of FC measurements between two subsets within each individual. As shown in Figure 4, with 400 time points, Pearson’s r, MGC statistics, and optimal scale exhibited a similar spatial distribution, with higher discriminability observed in the association cortex and lower discriminability in the sensorimotor and insular regions. Overall, FC measured by Pearson’s r displayed a higher whole-brain discriminability (0.983) than MGC statistics (0.938) and the optimal scale (0.922). At the parcel level, discriminabilities were 0.761 ± 0.090 for Pearson’s r, 0.704 ± 0.087 for MGC statistics, and 0.618 ± 0.072 for the optimal scale (Supplementary Table S1).

When the data increased to 800 time points per subset for each individual, the spatial patterns of reliability remained consistent, with enhanced discriminability indicating greater reliability over a 2-day test-retest measurement, particularly in the association cortex. Specifically, whole-brain discriminability improved to 0.997 for Pearson’s r, 0.987 for MGC statistics, and 0.993 for the optimal scale. Parcel-level discriminabilities also increased to 0.854 ± 0.090 for Pearson’s r, 0.789 ± 0.103 for MGC statistics, and 0.694 ± 0.091 for the optimal scale. These findings were replicated using Schaefer200 (Schaefer et al., 2018) parcellations, with detailed discriminability values presented in Supplementary Table S1 and Supplementary Figure S2.

### 3.3 Multiscale dependencies of homotopic connectivity

**Fig. 5.**
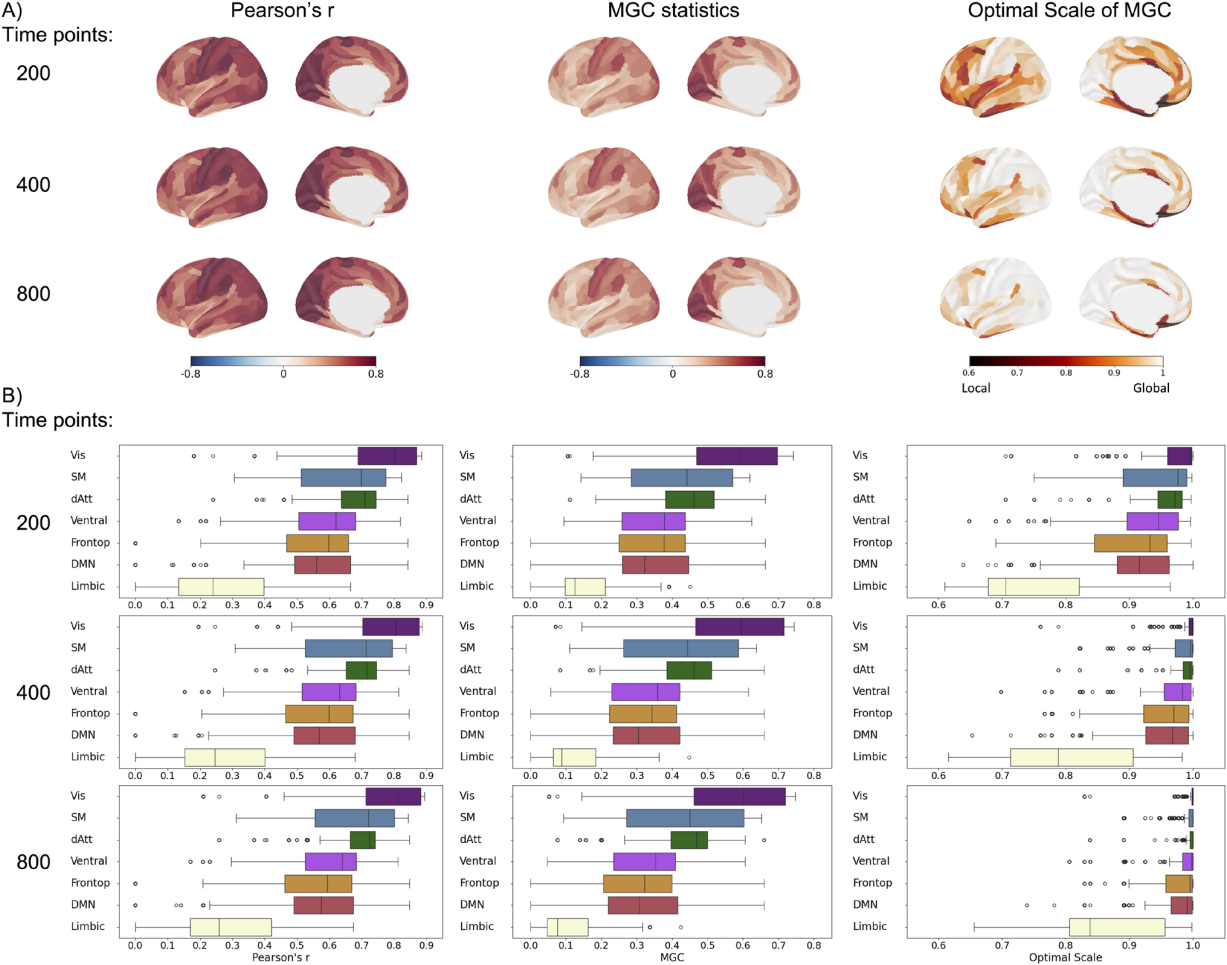
Homotopic connectivity measured by Pearson’s r, MGC statistics, and optimal scale across different data quantities (200, 400, and 800 time points per scan for each individual). (A) Group-averaged homotopic connectivity maps plotted on the left hemisphere and (B) Network-level connectivity values. The optimal scale of MGC in various acquisition durations showed a shift from local to global optimal scales as the duration increased.

Given the stability of homotopic FC between two hemispheres, Study 3 focused on homotopic connectivity to examine whether MGC captures FC beyond the linear Pearson’s r method in human and macaque subjects. As shown in Figure 5, we calculated homotopic connectivity across varying time points using Pearson’s r, MGC statistics, and the optimal scale. Across data with 200, 400, and 800 time points, the MGC-measured homotopic connectivity showed a spatial distribution similar to that observed with Pearson’s r. Both exhibited higher homotopic connectivity in posterior regions (e.g., visual, precuneus) and lower connectivity in anterior regions (e.g., frontal and insular cortex). The optimal scale of MGC revealed a shift from local to global optimal scales as the number of time points increased, suggesting that longer scan durations enabled the detection of dependencies sustained across broader scale ranges (Fig. 5A). Notably, the ventromedial prefrontal cortex (parcel OFC and area 25), medial cingulate region (33pr), language region (55b), angular gyrus (PSL), and hippocampus exhibited nonlinear relationships with relatively localized optimal scales even as the number of time points increased, indicating that dependencies in these regions may fluctuate or emerge over shorter intervals. At the network level (Fig. 5B), both Pearson’s r and MGC showed higher values on the visual network and the lowest value on the limbic network; the optimal scale maps further suggested that visual network connections exhibit global scale predominantly, while the limbic network dependencies may be more localized, indicating nonlinear connections (Fig. 5B).

### 3.4 Comparison across different repetition times (TRs) and an additional nonlinear method

To assess the impact of TR on FC estimates measured by Pearson’s r and MGC, we further calculated the homotopic FC using a subset of 85 subjects from the NKI-RS dataset who underwent resting-state scans at two different TRs (1400 ms and 645 ms). The spatial patterns of homotopic FC across TRs were highly similar at the group level (Supplementary Fig. S4, left panel) and relatively comparable within individuals for both Pearson’s r and MGC statistics (Supplementary Fig. S4, right panel). The agreement between two TRs at the individual level is high for both methods (discriminability: Pearson’s r = 0.977, MGC statistics = 0.979). In contrast, the optimal scale of MGC showed low similarity across TRs, likely reflecting its sensitivity to the temporal sampling rate determined by TR (Supplementary Fig. S4).

Additionally, we extended the homotopic FC analysis by including DTW as an additional nonlinear measure and compared it with Pearson’s r and MGC statistics (Supplementary Fig. S3). Overall, the scale of DTW increased with longer time series (Supplementary Fig. S3A, right). Its spatial patterns showed broadly similar to the other methods, though its similarities to Pearson’s r were lower than those between MGC statistics and Pearson’s r (Supplementary Fig. S3B, left). Notably, among the three methods, DTW exhibited the lowest test-retest reliability (discriminability: Pearson’s r = 0.935, MGC = 0.915, and DTW = 0.852 for 800 time points, Supplementary Fig. S3B).

**Fig. 6.**
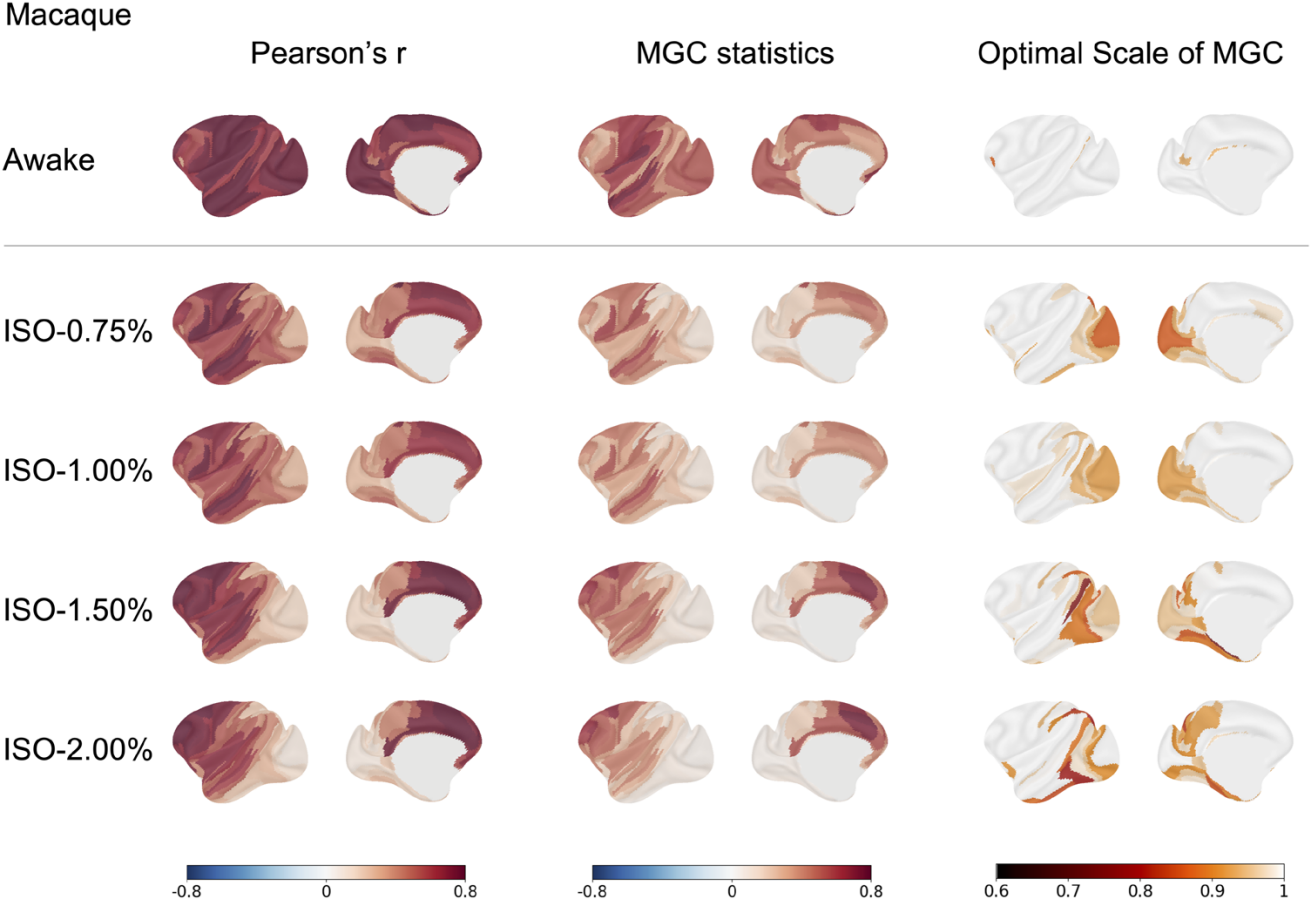
Macaque homotopic connectivity measured by Pearson’s r, MGC statistics, and the optimal scale of MGC across awake and anesthetized states at isoflurane concentrations of 0.75%, 1.00%, 1.50%, and 2.00%. The optimal scale of MGC in the macaque showed a shift from global to local as the isoflurane concentration increased.

In addition, we assessed homotopic connectivity in macaque monkeys across different anesthetized states with isoflurane concentrations of 0.75%, 1.00%, 1.50%, and 2.00%. As shown in Figure 6, the spatial pattern of homotopic connectivity measured by MGC statistics in macaques was closely aligned with Pearson’s r. Notably, from light to deep anesthetized states, the optimal scale decreased from global to local with increasing isoflurane concentrations, highlighting a prevalence of localized nonlinear temporal dependency under deeper anesthesia, particularly in visual areas. This trend was consistently observed across the anesthesia conditions in the other two macaque monkeys (Supplementary Fig. S5A and Supplementary Fig. S5B).

### 3.5 Pearson’s r and MGC captured similar brain-behavior associations

In Study 4, we aimed to assess whether Pearson’s r and MGC capture distinct brain-behavior associations by examining the effects of age and sex on homotopic connectivity using Pearson’s r and MGC. Both methods produced highly consistent age effects across the cortex, showing similar significant clusters (FDR-corrected p < 0.05) between the two methods (Supplementary Fig. S6, t-value map). A similar spatial pattern of the sex effect was observed, with no parcels retaining significance after FDR correction. No significant age or sex effect was detected for the optimal scale of MGC. These results indicated that both Pearson’s r and MGC were equally effective in capturing age-related changes in functional homotopic connectivity—MGC provides no incremental advantage over Pearson’s r in detecting brain-behavior associations.

## 4 Discussion

The present study systematically compared FC measurements obtained using the traditional Pearson’s correlation coefficient (Pearson’s r) and recently developed Multiscale Graph Correlation (MGC) across diverse datasets, varying data quantities, distinct experimental conditions, as well as in capturing brain-behavior relationships. To our knowledge, this is the first study to systematically investigate and compare MGC with traditional Pearson’s r-based FC. Overall, Pearson’s r and MGC captured similar FC profiles across the cortex. Reliability is generally higher using Pearson’s r compared to MGC, and both methods showed improved reliability as the amount of data increased. Notably, functional interactions within networks are predominantly of global scale and time-invariant across the entire fMRI signals, making Pearson’s r a sufficient and efficient metric for most applications. However, weaker, between-network interactions and altered physiological states (e.g., under anesthesia) exhibited more localized or scale-specific nonlinear dependencies that MGC was uniquely positioned to identify, particularly through its ability to determine the optimal scale of dependencies.

Across datasets, Pearson’s r effectively captured strong, stable connections, particularly within-network connectivity. Specifically, FC within networks using Pearson’s r and MGC were substantially similar, with MGC’s optimal scale often categorized as ‘global’, suggesting predominantly near-linear relationships. In other words, within-network FC is well-suited to the linear assumptions of Pearson’s r, as these connections often reflect sustained, ongoing synchronization that remains stable across the duration of a scan. Conversely, between-network connections exhibited weaker Pearson’s r values and more localized optimal scales under MGC, suggesting more complex nonlinear interactions over time. While Pearson’s r often identifies these connections as weaker or even negative when the global signal is removed in preprocessing, previous work has shown that these weaker connections also mirror the functional organization of their stronger counterparts, reflecting the principles of “common friends, common enemies” (Nenning et al., 2023). In the present study, by identifying local optimal scales, MGC revealed that these inter-network interactions may not be uniformly consistent across time points, indicating nonlinear dependency between these brain regions, which might also reflect the presence of dynamic, time-varying connections over shorter time scales (Allen et al., 2014; Calhoun et al., 2014; Chang & Glover, 2010). Moreover, findings from dynamic FC studies suggest that interactions between networks are not constant but fluctuate over time (Chang & Glover, 2010; Hutchison et al., 2013). Given that the ‘optimal scale’ estimated by MGC represents the scale (i.e., the number of time point samples) that achieves the strongest dependency between brain regions, it can be loosely interpreted as a data-driven window size for further investigation into temporal dynamic FC analysis. In the present study, this is supported by the high similarity of spatial patterns observed between the MGC statistic and the dynamic time warping method (Supplementary Fig. S3). Together, these findings highlight the importance of employing alternative statistical dependency measures to capture the nonlinear local changes in the temporal dependency of FC.

Our analysis also highlighted the importance of data quantity in capturing stable and reliable connectivity patterns. Both Pearson’s r and MGC benefited substantially from larger datasets. As the amount of data increased, the strengths of connections became more pronounced, particularly for strong connections, which tend to be linear relationships. With sufficient data, the distinction between strong and weak connections becomes clearer, with stronger connections revealing more global associations. This is consistent with previous studies suggesting that larger amounts of fMRI data are required to characterize stable connections between regions, especially at the individual level (Finn et al., 2015; Gratton et al., 2020). When comparing the reliability of MGC and Pearson’s r, our results indicated that Pearson’s r generally provided higher reliability across datasets. This difference was especially apparent with limited data, where MGC’s reliability was lower, likely due to the increased sensitivity of its optimal scale to noise in such conditions. However, the reliability of MGC improved as data quantity increased, indicating its potential for capturing more nuanced dependencies when sufficient data are available. These results suggest that Pearson’s correlation remains the more robust method for reliably measuring strong linear connections, particularly in studies with limited data.

MGC demonstrates its value in specific contexts, particularly in detecting weaker connections and identifying their optimal scale under different scan conditions. In our analysis of macaque data, which examined arousal differences between awake and different anesthetized states, MGC captured transitions in connectivity strength and revealed more local homotopic connectivity during anesthesia. Under anesthesia, brain activity is often intermittent, alternating between periods of suppressed activity and relative bursts, resulting in nonlinear interactions. This phenomenon has also been observed in humans, where the brain alternates between active and quiescent states (Sirmpilatze et al., 2022). These findings highlight the unique advantage of MGC in uncovering the scale of FC, particularly in altered states of brain activity where nonlinear interactions dominate and dependencies may vary over time. However, despite its sensitivity to these dynamic and nonlinear dependencies, MGC was not superior to Pearson’s r in brain-behavior association analyses, suggesting that the predominant relationships underlying brain-behavior links may remain linear or near-linear in nature.

Overall, this study demonstrated that Pearson’s correlation is generally sufficient in resting-state fMRI for capturing strong and robust connections, particularly within-network FC, which tends to be linear and fairly constant over time. Yet, between-network interactions and certain altered physiological states (like deeper anesthesia) may display more complex time-varying dependencies, for which MGC’s capacity to detect local, nonlinear effects can be revealing. Consequently, while Pearson’s r remains an efficient, highly interpretable metric for many standard applications, MGC can provide additional insights for phenomena suspected to be dynamic and less stable or linear. Given the increased computational cost of MGC, researchers should carefully weigh these trade-offs but consider more advanced methods when the full spectrum of dynamic or nonlinear dependencies is of scientific interest.

## Supplementary Material

Supplementary Material

## Data Availability

The Human Connectome Project datasets are publicly available from the HCP Young Adult release (https://www.humanconnectome.org/study/hcp-young-adult). The Nathan Kline Institute Rockland Sample datasets are available through the Database of the International Neuroimaging Data-sharing Initiative (INDI). https://fcon_1000.projects.nitrc.org/indi/enhanced/ The code for MGC calculation is provided by Python package ‘hyppo’ at https://hyppo.neurodata.io/api/generated/hyppo.time_series.mgcx. The code for our analyses in our study is provided at https://github.com/HechengJin0/MGC_paper
