# Supplementary material for: Is Pearson’s correlation coefficient enough for functional connectivity in fMRI?
Source: Imaging Neurosci (Camb). 2025 Dec 8;3:IMAG.a.1052. doi: 10.1162/IMAG.a.1052 (PMC12687289; doi:10.1162/IMAG.a.1052)
Supplement: Supplementary Material [file IMAG.a.1052_supp.pdf]

Supplementary

Is Pearson's correlation coefficient enough for functional connectivity in fMRI?

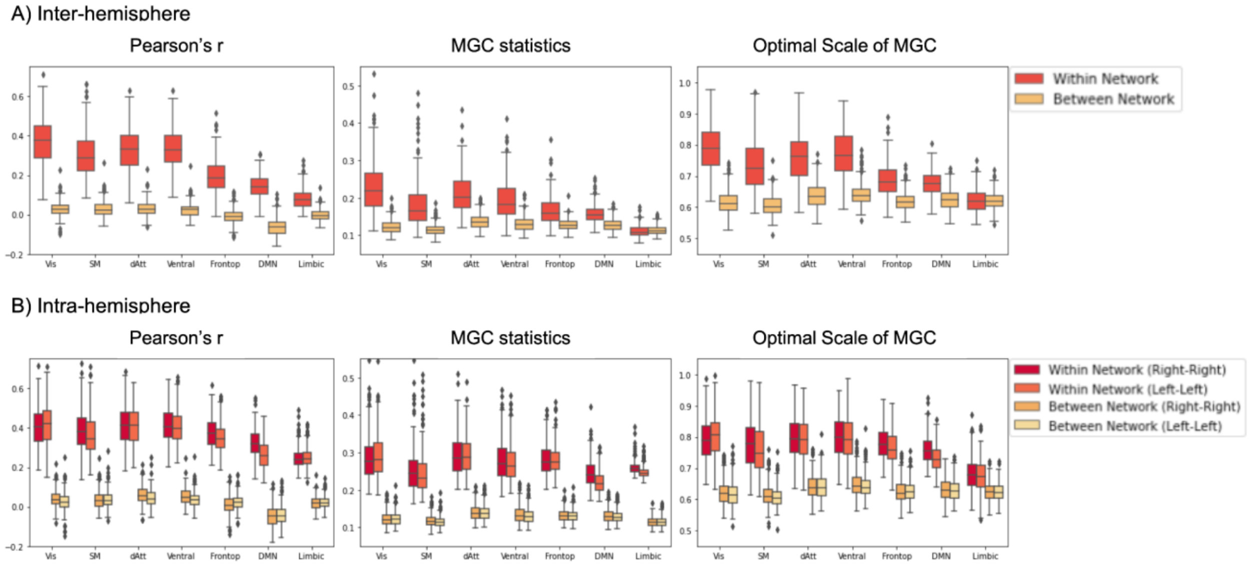

Figure S1. Functional connectivity measured by MGC statistics and optimal scale values on 7 networks: Both inter- and intra-hemispheric FC showed more global associations (higher optimal scale) within networks than between networks.

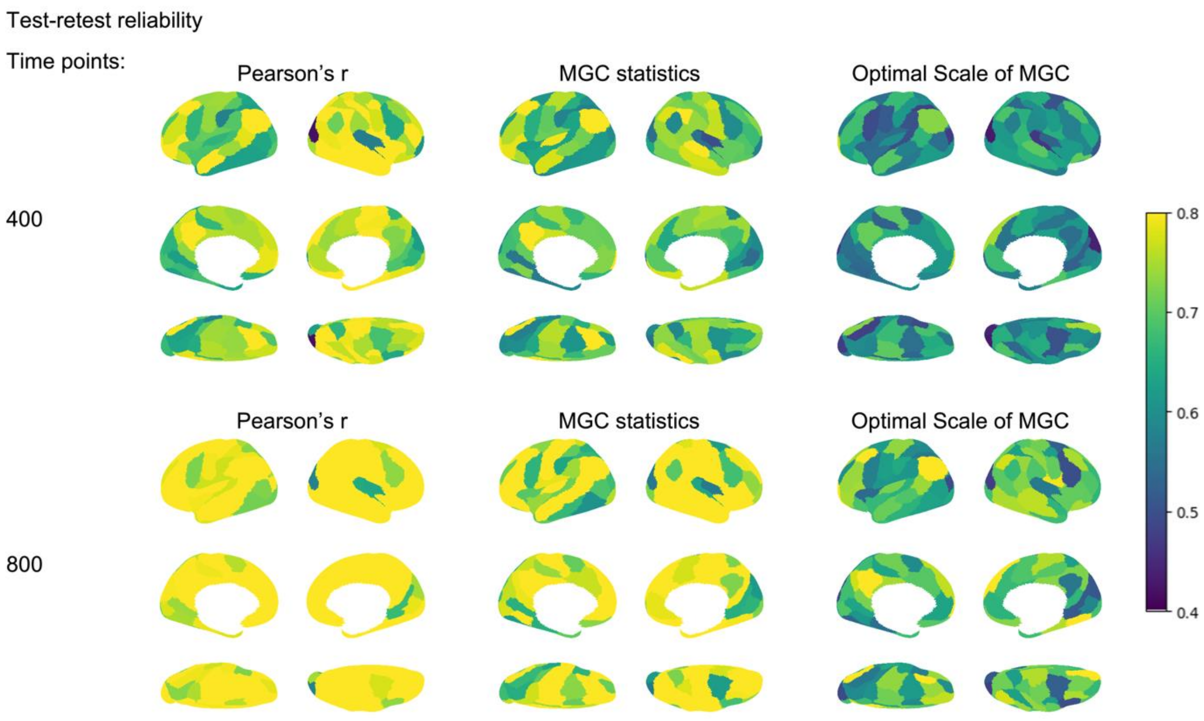

Figure S2. Test-retest reliability for 400 time points (averaging FC across 4 scans, each consisting of 200 time points) and 800 time points (averaging FC across 4 scans, each consisting of 200 time points) on the Schaefer200 (Schaefer et al., 2018) parcellations.

A)

Glasser et al. (2016) atlas with 360 parcels Discriminability				
Acquisition Time		Pearson's r	MGC statistics	Optimal Scale of MGC
400 Time points	Individual parcel	0.7613 ± 0.0901	0.7036 ± 0.0868	0.6178 ± 0.0724
	Whole-brain	0.9833	0.9376	0.9224
800 Time points	Individual parcel	0.8539 ± 0.0902	0.7887 ± 0.1033	0.6944 ± 0.0905
	Whole-brain	0.9968	0.9871	0.9934

B)

Schaefer et al. (2018) atlas with 200 parcels Discriminability				
Acquisition Time		Pearson's r	MGC statistics	Optimal Scale of MGC
400 Time points	Individual parcel	0.7414 ± 0.0792	0.6923 ± 0.0771	0.5977 ± 0.0668
	Whole-brain	0.9589	0.9164	0.8615
800 Time points	Individual parcel	0.8452 ± 0.0784	0.7807 ± 0.0913	0.6745 ± 0.0803
	Whole-brain	0.9897	0.9727	0.9790

Table S1. Test-retest reliability of individual parcels and whole-brain values for 400 time points and 800 time points on the Schaefer200 (Schaefer et al., 2018) and Glasser (Glasser et al., 2016) parcellations.

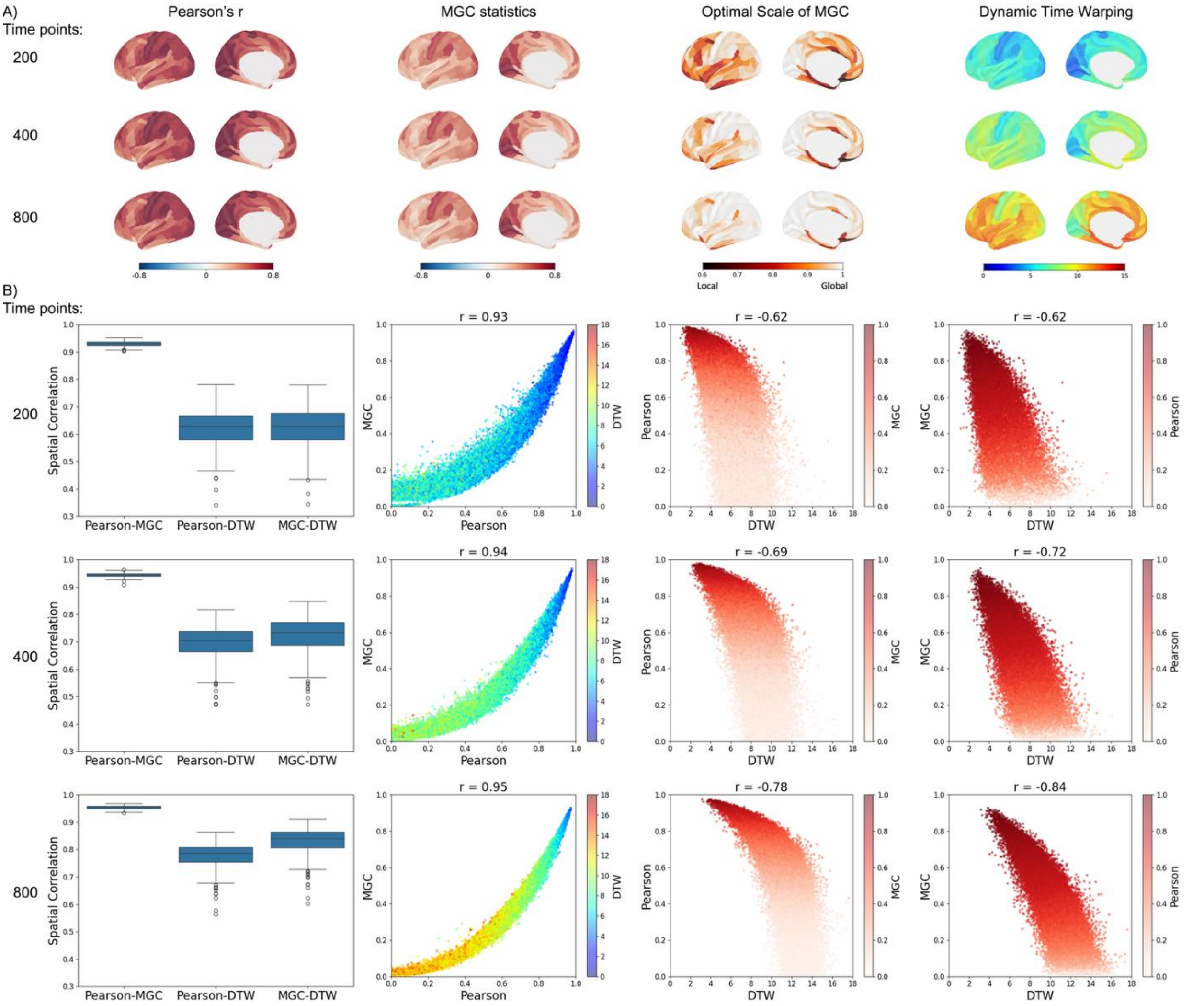

Figure S3. Homotopic connectivity measured by Pearson's r, MGC statistics, optimal scale, and dynamic time warping across different data quantities (200, 400, and 800 time points per scan for each individual). ***A)*** *Group-averaged homotopic connectivity maps plotted on the left hemisphere.* ***B)*** *Spatial similarity of homotopic connectivity measured by the absolute value of Pearson's correlation coefficient at the individual level and scatter plots across methods. Test-retest reliability (discriminability): Pearson's r=0.863, MGC=0.840, DTW=0.747 for 200 time points; Pearson's r=0.920, MGC=0.898, DTW=0.807 for 400 time points; and Pearson's r=0.935, MGC=0.915, DTW=0.852 for 800 time points.* ***Abbreviations****: MGC = Multiscale Graph Correlation statistics; DTW = Dynamic Time Warping.*

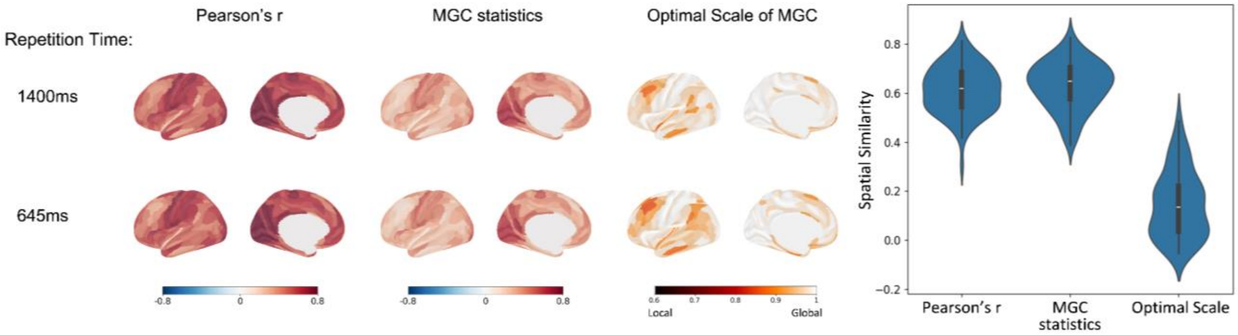

Figure S4. Homotopic connectivity across different repetition times (TR = 1400 ms and 645 ms, NKI-RS sample, N=85), measured by Pearson's r, MGC statistics, and the optimal scale of MGC. Group-averaged homotopic connectivity was plotted on the left cortical surface. The violin plots show the spatial similarity of homotopic connectivity within individuals between TR conditions. Spatial similarity: Pearson's r=0.614 ± 0.096, MGC statistics=0.631 ± 0.096.

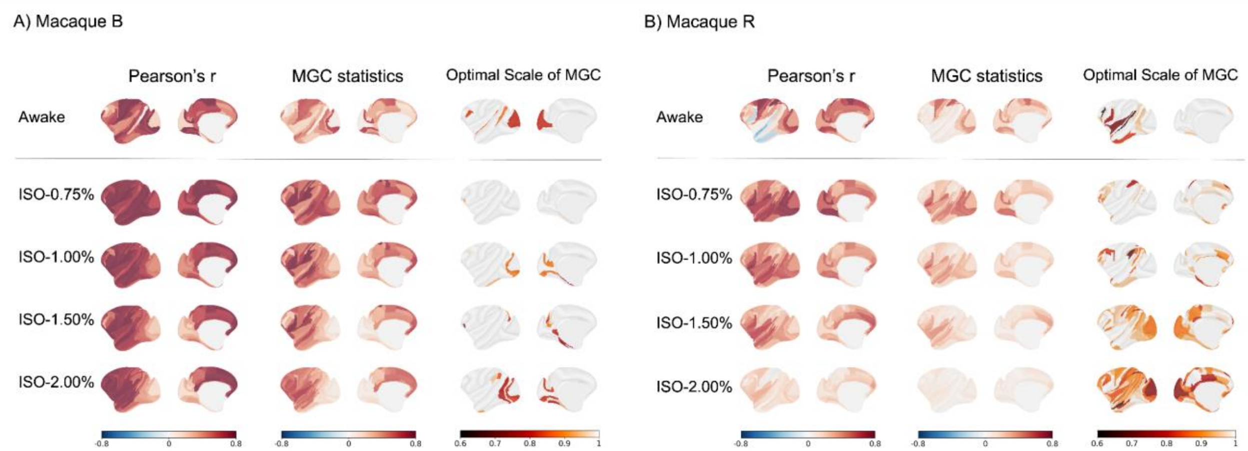

Figure S5. Macaque B and macaque R homotopic connectivity measured by Pearson's r, MGC statistics, and the optimal scale of MGC across the awake state and anesthetized at 0.75%, 1.00%, 1.50%, and 2.00% isoflurane concentrations. The optimal scale of MGC in the macaque showed a shift from global to local optimal scales as the isoflurane concentration increased.

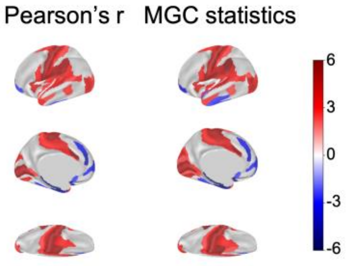

Figure S6. Impact of age (sex-adjusted) on developmental brain homotopic connectivity measurements. Brain maps displaying t-values (showing parcels with FDR-corrected $p < 0.05$) for Pearson's r measurements and MGC statistics.

	Runtime (ms)		
Time Points	Pearson's r	MGC	Dynamic Time Warping
200	0.7 ± 0.1	122.0 ± 3.9	413.6 ± 5.1
400	0.7 ± 0.1	402.5 ± 15.0	1689.8 ± 8.8
800	0.7 ± 0.1	1623.7 ± 59.6	6855.1 ± 29.8

Table S2. Runtime comparison of Pearson's r, MGC, and Dynamic Time Warping (DTW) across varying numbers of time point samples. All computations were performed using a single CPU, averaged over 100 repeated runs.
